# Effects of study design and allocation on self-reported alcohol consumption: randomized trial

**DOI:** 10.1186/s13063-015-0642-0

**Published:** 2015-03-28

**Authors:** Kypros Kypri, Amanda Wilson, John Attia, Paschal J Sheeran, Jim McCambridge

**Affiliations:** Centre for Clinical Epidemiology and Biostatistics, School of Medicine and Public Health, University of Newcastle, Callaghan, NSW 2308 Australia; Department of Psychology, University of North Carolina at Chapel Hill, Chapel Hill, NC USA; Faculty of Public Health & Policy, London School of Hygiene & Tropical Medicine, London, UK

**Keywords:** Alcohol, Allocation, Behaviour, Hawthorne effect, Internet, Placebo effect, Randomized controlled trial, Reactivity, Students, University

## Abstract

**Background:**

What participants think about the nature of a study might affect their behaviour and bias findings. We tested two hypotheses: (1) participants told they were in an intervention trial would report lower alcohol consumption at follow-up than those told they were in a cohort study; (2) participants told they were in the intervention group in a trial would have lower alcohol consumption at follow-up than those told they were in the control group.

**Methods:**

Students from four universities (*N* = 72,903) were invited to participate in a ‘research project on student drinking’. Of 10,415 respondents, 6,788 were moderate to heavy drinkers and were randomized. Group A (‘cohort’) were informed their drinking would be assessed at baseline and again in one month. Group B (‘control’) were told the study was an intervention trial and they were in the control group. Group C (‘intervention’) were told the study was an intervention trial and they were to receive the intervention. All were assessed and directed to read identical online alcohol education material. Whether and how long they accessed the material were recorded. One month later, alcohol intake was reassessed.

**Results:**

In relation to hypothesis 1, there were no differences between the groups on the prespecified outcome measures. In relation to hypothesis 2, there were no differences though all point estimates were in the hypothesized direction (that is, ‘intervention’ < ‘control’). The ‘cohort’ and ‘control’ groups accessed the material to a similar extent (59% versus 57%) while the ‘intervention’ group were more likely to access it (78%) and to read it for longer (median 35 s (25th and 75th percentiles: 6, 97) versus medians of 7 s (0, 28) and 8 s (4, 42) for the ‘cohort’ and ‘control’ groups, respectively).

**Conclusions:**

Although the context given to the research participants significantly influenced access to the online information and reading time, this did not translate into any effect on drinking behaviour, for either hypothesis. This might be because of failure in the experimental paradigm or the possibility of weaker effects using the online approach.

**Trial registration:**

Australian New Zealand Clinical Trials Registry ACTRN12610000846022

## Background

Social science is increasingly relied upon to develop behavioural interventions to improve health, for example, encouraging smoking cessation, reducing alcohol consumption, improving diet and increasing physical activity. Such research typically involves monitoring participants’ behaviour and trials involve randomization of participants to intervention or control groups. Despite awareness of the potential for both trial-specific processes [1] and more generic research participation effects [2] to affect behaviour in ways that might bias estimates of intervention effects, there has been little systematic study of these issues.

There have been concerns about impacts of various aspects of taking part in research studies since the discovery of pre-test sensitization [3]. Research studies are unusual contexts, and people might react in unexpected ways to what we invite them to do [2]. Such problems might afflict all study designs, including the most rigorous available for causal inference. Research participation effects occurring before randomization may interact with evaluated interventions, thereby introducing bias into effect estimates in randomized trials [4]. Similarly, participants might react in unintended ways to allocation in randomized trials, biasing effect estimates in different ways [5]. We have previously offered the construct of research participation effects to guide empirical studies and advocated adopting a more participant-centred view of the research process [2].

As a consequence of the observation that patients bring hopes and expectations when they enrol in clinical trials, the design of trials has had to adapt to accommodate and account for patient preferences [1]. In addition, it has been proposed that the uncertainty inherent in studies involving random allocation might lead control group participants to either try harder (‘compensatory rivalry’) or give up (‘resentful demoralization’) [6]. Cook and Campbell [6] postulated these direct effects of study design and allocation or randomization in 1979, yet their existence has not been verified. There are equally plausible psychological explanations for individuals or clusters assigned to intervention behaving in ways that bias effect estimates, for example, if those randomized have a strong emotional investment in receiving a novel, potentially beneficial, intervention, they may do better than they would outside the context of a research trial.

In response to this gap in the research literature regarding the impact of research conditions on the behaviour of study participants, and the inferences researchers wish to draw from observational or intervention studies, we have reviewed the methodological literature in several fields (for example, [7,8]) and developed a conceptual framework to advance multidisciplinary understanding of bias in the study of human behaviour [2]. A priority identified in that work is the investigation of the effects of randomization posited by Cook and Campbell in 1979 [6].

We were interested in the possibility that awareness of being in an intervention trial, and thus of being randomly allocated, might lead participants to focus their attention on the behaviour of interest. They may, as a consequence, be more alert to the possibility of change, and develop expectations about change, to a greater extent than participants in a study in which the behaviour of interest is merely being measured. Similarly, participants who are told they have been randomly allocated to the intervention arm of a behaviour change trial might be more likely to focus on that behaviour and change it than participants allocated to a control group, who have not been given an implicit or explicit message that change may occur.

In this study, we recruited university students by email to visit a web-based description of the project and to answer questions about their drinking. The experimental manipulations consisted of what students were told about the purpose and nature of the study. To isolate the effects of this manipulation, a page of educational material, expected to be ineffective in modifying drinking behaviour (education alone is shown to be ineffective in reducing college student drinking [9]), and chosen so as to be compatible with all three study descriptions given to the study groups, was presented to all participants.

We designed an experiment to test two hypotheses:

1. That knowledge of participation in an intervention trial in comparison with a cohort (that is, an observational) study alone would reduce subsequent self-reported drinking after one month.
2. That knowledge of allocation to an intervention condition in comparison with a control condition in a randomized trial would reduce subsequent self-reported drinking after one month.

## Methods

### Design

**Figure 1:**
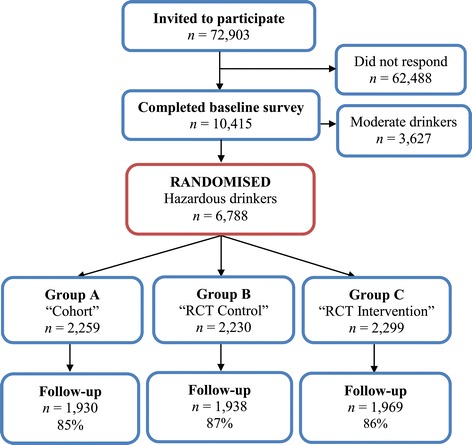
**Trial flow chart.**

We undertook a three-arm randomized trial (Figure 1). The experimental manipulation consisted of what participants were told about the design of the study: group A (‘cohort’) were told they were in a cohort (that is, longitudinal) study, group B (‘control’) that they were in an intervention trial and had been randomly assigned to the control group, and group C (‘intervention’) that they were in an intervention trial and had been randomly assigned to the intervention group [10]. In fact, all participants received identical information about the health consequences of alcohol consumption. A copy of the specific wording used to effect the experimental manipulation and the health information provided to participants has been published [10] along with the trial protocol [10].

### Setting

The setting was four public universities in New Zealand and recruitment and randomization were undertaken in two waves; recruitment at one university in 2010 provided adjustment to the sample size estimate (described in the protocol [10]) for a second stage involving three further universities in 2011.

### Procedure

All students were invited by email to participate in an online ‘research project on student drinking’ using well established procedures [11]. Students were informed that, ‘The study involves the completion of two short web surveys each one month apart,’ and were asked to click on a hyperlink to the study website and information form. In the invitation email and study information page, students were informed that they would be entered into a draw to win an Apple iPad if they completed the baseline and follow-up questionnaires. A reminder message was sent two weeks later. Participants provided consent to participate by clicking on the link to complete the first survey (see ethical considerations).

### Pilot research

For the experimental manipulation to work it was important that the alcohol health information appeared credible to all three groups, and we developed text to meet these requirements. Our pilot research, described in the trial protocol [10], showed that the alcohol health information and the way it was presented to participants did appear credible to all three groups. Students were unaware of the true nature of the study and participants allocated to all three groups found the provision of alcohol education material credible, that is, that *Alcohol: The Basics* served simultaneously as information one might be unsurprised to receive as a participant in a cohort study, or as a participant in the control condition of a randomized trial of an alcohol intervention, or as the recipient of the intervention in the intervention arm of the trial.

### Screening

The baseline survey was comprised of the three questions from the Alcohol Use Disorders Identification Test - Consumption (AUDIT-C) subscale [12] (items 1 to 3 in this list with scoring in square brackets):

1. How often do you have a drink containing alcohol? (Response options: never or almost never [0]; less than once a month [1]; once a month [1]; once every two weeks [2]; once a week [2]; two or three times a week [3]; four or five times a week [4]; six or seven times a week [4]).
2. How many standard drinks containing alcohol do you have on a typical day when you are drinking (Please refer to the standard drinks guide on the left)? (Response options: 1 [0], 2 [0], 3 [1], 4 [1], 5 [2], 6 [2], 7 [3], 8 [3], 9 [3], 10 [4] … 24, 25–29, 30–34, 35–39, 40–49, 50 or more [all response options > 10 scored as 4]).
3. How often do you have six or more standard drinks on one occasion? (Response options: never [0]; once or twice a year [1]; less than monthly [1]; monthly [2]; weekly [3]; daily or almost daily [4]).

These were followed by three further questions from a previous study [13] to enhance face validity:

4. What is your favourite type of drink? (Respondents could choose one of: beer, wine, spirits, pre-mixed or ready to drink spirits, cider, other, none).
5. Where do you most like to drink? (Respondents could choose one of: at home or my hall of residence; at friends’ houses; in pubs and bars; at my parents’ home; at the beach; in parks; other places).
6. Which of the following do you do in your spare time? (Respondents could select any combination of: listen to music (for example, CDs or records) at home; listen to live music (for example, bands or DJs); visual arts (for example, painting, sculpture); reading; performing arts (for example, playing music, theatre); social sport; competitive sport; religious activities; outdoor activities (for example, tramping, skiing, fishing); political and community activities; other (please state)).

Those whose answers indicated a low-risk drinking (score < 4 in the AUDIT-C subscale [12]) were not eligible for the trial and were thanked for participating and provided with a link to *Alcohol: The Basics*, a page containing information about effects of alcohol, safe drinking levels, and problems associated with drinking, such as drink-driving.

### Randomization

Respondents with a moderate to high level of alcohol intake (indicated by a score of 4 or more on the AUDIT-C subscale [12]) were randomized without their knowledge to one of three conditions (A, B or C). Randomization was effected by computer using a random number generator, with a 1:1:1 allocation ratio. Participants were not informed that they were participating in a randomized study and, because randomization was computerized, the research team did not know which group each participant was assigned to until after outcomes were assessed. There was thus no opportunity for randomization to be subverted.

### Ethical considerations

Deception was used to ensure that participants were blind to the true nature of the study, as any such knowledge would self-evidently interfere with hypothesis testing [14]. All participants were offered debriefing upon completion of the study [15]. Ethical approval to conduct the pilot research was granted by the University of Newcastle Human Research Ethics Committee (protocol number H-2010-1243) and approval to conduct the trial was granted by the University of Otago Research Ethics Committee (Protocol number 10/148).

### Interventions

Participants in the three experimental groups were presented with the opportunity to access the *Alcohol: The Basics* material via a hyperlink. This material was developed according to our understanding of the ineffectiveness of such information in promoting behaviour change, and there were identical levels of encouragement to read the alcohol health information in each condition. The differences between groups existed solely in the way the study was described to participants, namely, in what students were told was the design of the study (cohort or trial), along with their allocation status (control group or intervention group) if randomized to groups B or C.

### Outcome measurement

At baseline, participants were each advised that they would be emailed a link to another survey in a month’s time. The second survey contained the three AUDIT-C items used at baseline (shown above), and five further questions:

4. On how many days did you have a hangover in the last 4 weeks?
5. What would you say was the design of the study? (Response options: two separate surveys of student drinking; following up a group of student drinkers over time; other (please explain how you perceived the study design)).
6. After completing the survey last month did you think more about your drinking? (Response options: no, yes).
7. After completing the survey last month did you change your drinking? (Response options: my drinking did not change, my drinking decreased, my drinking increased).
8. Respondents who reported decreased drinking in Q7 were asked, ‘Was this due to taking part in the survey?’ (Response options: no, yes).

Participants were then sent to a ‘thank you’ page and offered the opportunity to type comments about the study into a text box.

The primary outcome measures were scores on the first four questions: the frequency of drinking, quantity of alcohol consumption per typical drinking occasion (in standard drinks; 10 g ethanol), volume of alcohol consumed in the preceding four weeks (the product of frequency by quantity of alcohol consumption per typical drinking occasion), and the incidence of hangovers in the same period.

### Analysis

As per the study protocol [10], the ‘control’ and ‘intervention’ groups were combined for comparison against the ‘cohort’ group to test hypothesis 1, and the ‘control’ and ‘intervention’ groups were compared with each other to test hypothesis 2. Reported alcohol consumption was analyzed using negative binomial regression, with baseline AUDIT-C subscale score as a covariate [16]. Hangover incidence was analyzed using logistic regression. Results are presented as risk ratios and odds ratios. Participants were analyzed in the group to which they were randomized. We measured reading duration as the time elapsed while the *Alcohol: The Basics* page was open, as an indicator of the degree of engagement with the material. This was based on the expectation that participants who thought they were in an intervention trial would take more interest in the study than participants who thought they were in a cohort study, while those told they had been assigned to the intervention group would engage most.

We also conducted three sets of *post-hoc* exploratory analyses: (1) comparing groups A versus C in light of the possibility of failure in the experimental manipulation (discussed next); (2) adjusting for age on the assumption that younger participants might be more or less susceptible to the hypothesized effects (for example, study participants have been shown to vary by age in their suggestibility [17]); and (3) comparing subgroups with AUDIT-C subscale scores of 4 to 6 versus 7 to 12 on the assumption that the heaviest drinkers might be less susceptible to the hypothesized effects; a total of 32 additional tests.

The sample size estimation procedure is presented in the trial protocol [10]. All data analysis was undertaken in Stata 12.0.

## Results

Figure 1 shows that 6,788 individuals were randomized and that percentages followed up were high and similar across the three experimental groups.

### Planned analyses

Table 1 shows the demographic characteristics and AUDIT-C scores of the participants, and that the three experimental groups were similar at baseline. The ages of participants varied from 17 to 61 years, reflecting inclusion of all students enrolled at the four universities.

**Table 1:** **Participant characteristics at baseline**

	**Experimental group**		
	**A (cohort)** ***n*** **= 1930**	**B (control)** ***n*** **= 1938**	**C (intervention)** ***n*** **= 1969**
Women, *n* (%)	1177 (61%)	1176 (61%)	1153 (59%)
Age in years: mean (standard deviation)	22.9 (6.9)	22.6 (6.2)	22.7 (6.4)
AUDIT-C subscale score: mean (standard deviation)	6.6 (2.0)	6.6 (2.1)	6.6 (2.0)

Table 2 shows effect ratios for the four prespecified outcomes pertaining to the two hypotheses. All but one of the estimates was in the hypothesized direction but none was statistically significant (*P* < 0.05).

**Table 2:** **Effect estimates**

	**Effect ratio (95% CI)**	
	**Hypothesis 1 (control and intervention)/cohort**	**Hypothesis 2 intervention/control**
1. Frequency of drinking	**0.98** (0.94 to 1.02) *P* = 0.27	**0.99** (0.94 to 1.03) *P* = 0.54
2. Typical occasion quantity	**0.98** (0.95 to 1.02) *P* = 0.31	**0.99** (0.96 to 1.03) *P* = 0.63
3. Overall volume consumed	**0.96** (0.93 to 1.01) *P* = 0.09	**0.97** (0.93 to 1.02) *P* = 0.25
4. Frequency of hangover	**1.02** (0.96 to 1.09) *P* = 0.48	**0.94** (0.88 to 1.01) *P* = 0.09

All outcomes had a reference period of the preceding 4 weeks.

Table 3 shows the percentage in each group who clicked on the hyperlink to visit the *Alcohol: The Basics* webpage and the median number of seconds spent with the webpage open. There was no difference between the ‘cohort’ and ‘control’ groups on either variable, indicating possible experimental manipulation. The ‘intervention’ group had a higher percentage (78%) who opened the page than the ‘cohort’ (59%, *P* < 0.001) and ‘control’ (57%, *P* < 0.001) groups, and they spent substantially longer (median of 35 seconds versus 7 and 8 seconds respectively, *P* < 0.001 for both comparisons) with the page open.

**Table 3:** **Access to the educational material and reading time in each experimental group**

	**Cohort (A)**	**Control (B)**	**Intervention (C)**
Clicked on *Alcohol: The Basics* hyperlink	59%	57%	78%
Median time spent on *Alcohol: The Basics*, in seconds (25th, 75th percentiles)	7 (0, 28)	8 (4, 42)	35 (6, 87)

### Exploratory analyses

#### ‘Cohort’ versus ‘Intervention’ (A versus C)

Given the lack of effect in relation to hypothesis 1, and the possibility of failure to operationalize the intended experimental manipulation (see also Table 4 row 1 and Discussion section), we sought to maximize the contrast by comparing the ‘cohort’ and ‘intervention’ groups on the four primary outcomes. Effect ratios for all four outcomes were in the hypothesized direction (ratios < 1) but only one of them (volume consumed, risk ratio = 0.95, *P* = 0.04) had a *P* < 0.05 which should be interpreted in light of the multiple tests performed, and the exploratory nature of these analyses.

**Table 4:** **Participant perceptions of the study design and effects on their drinking**

	**A (** ***n*** **= 1930)**	**B (** ***n*** **= 1938)**	**C (** ***n*** **= 1969)**	**Total (** ***N*** **= 5837)**
**What would you say was the design of the study?**				
Two separate surveys of student drinking	323 (17%)	322 (17%)	290 (15%)	935 (16%)
Following up a group of student drinkers over time	1,523 (79%)	1,515 (78%)	1,550 (79%)	4,588 (79%)
Other	84 (4.4%)	101 (5.2%)	129 (6.6%)	314 (5.4%)
**After completing the survey last month did you think more about your drinking?**				
No	1357 (70%)	1407 (73%)	1357 (69%)	4121 (71%)
Yes	455 (24%)	424 (22%)	500 (25%)	1379 (24%)
Don’t know	118 (6.1%)	107 (5.5%)	112 (5.7%)	337 (5.8%)
**After completing the survey last month did you change your drinking?**				
My drinking did not change	1493 (77%)	1535 (79%)	1532 (78%)	4560 (78%)
My drinking decreased	358 (19%)	317 (16%)	363 (18%)	1038 (18%)
My drinking increased	79 (4.1%)	86 (4.4%)	74 (3.8%)	239 (4.1%)
**Was this due to taking part in the survey?** (Asked of 1038 participants who said their drinking decreased)				
No	276 (77%)	251 (79%)	288 (79%)	815 (79%)
Yes	82 (23%)	66 (21%)	75 (21%)	223 (21%)

#### Adjustment by age

The results relating to the two primary hypotheses were similar after adjustment for the age of participants, with effect ratios varying from 0.94 to 1.02 and none of them with *P* < 0.05.

#### Drinking subgroups

The results relating to the two primary hypotheses were similar for participants who scored 4 to 6 versus 7 to 12 on the AUDIT-C subscale, with effect ratios varying from 0.93 to 1.14 and one of them with a *P* < 0.05 (hypothesis 1: risk ratio = 1.14 for hangover, *P* = 0.02).

Table 4 presents a summary of responses to the four questions concerning participants’ trial experiences. The results show that the majority of respondents judged the study design to involve repeated measurements but not intervention. A quarter of participants indicated that they thought more about their drinking after completing the baseline survey, and 18% thought that their drinking decreased in that period. About 21% attributed the decrease to completing the baseline survey. Importantly, there were was little variation between experimental groups in responses to these questions.

## Discussion

There were no differences between the groups (‘cohort’ versus ‘control’ and ‘intervention’; or ‘control’ versus ‘intervention’) in drinking one month after baseline as a function of what participants were given to believe about the study. Accordingly, neither of the null hypotheses can be rejected. There was a large difference in the percentage of the ‘intervention’ group who clicked on the alcohol information, and in the amount of time spent viewing that information, relative to the other groups, suggesting that the ‘intervention’ group treated the alcohol information in the way intended, as an intervention. There was no difference between the ‘cohort’ and ‘control’ groups in accessing of the alcohol information, suggesting the possibility of failure in the experimental manipulation, particularly as this measure was primarily designed as a manipulation check and was not specified *a priori* as an outcome measure.

Strengths of the study include the low potential for selection bias, given the random allocation of participants and similarity of the groups at baseline. The use of automated electronic data collection minimized or eliminated certain types of information bias (for example, from data entry), detection bias, and performance bias, that is, systematic variation in the treatment of groups other than that intended in the experimental manipulation. Attrition was low and similar across the groups. Adherence to prespecified outcomes and analytic procedures protects against reporting bias.

Limitations arise from possible bias in self-reporting of alcohol consumption. Objective measurement of alcohol consumption using direct observation or breath alcohol analysis was not feasible, and blood biomarkers (for example, gamma-glutamyl transferase) are not sensitive [18] to the episodic heavy consumption characteristic of this population group [13], even if it were feasible to test for them on the scale necessary to detect the small effects we hypothesized. While self-report of alcohol consumption might be considered a sensitive behaviour fairly reliably reported in conditions of confidential, computerized reporting [19] as applied in this study, it remains possible that the three groups varied in how much they misreported their drinking. Much study of the validity of self-reported drinking has taken place in treatment contexts (for example, [18]), and findings might not generalize to nontreatment populations [20]. It is unclear whether any such misreporting would bias effect estimates towards or away from the null hypothesis but it is plausible that participants given to believe they were in an intervention trial (groups B and C) would under-report their drinking more than those in the ‘cohort’ group, with participants who thought they were receiving intervention (group C) under-reporting most. Such bias might account for the fact that almost all of the effect ratios were in the hypothesized direction; however, given the large sample size and lack of statistical significance, these effects, if present, must be very small. Alternatively, reporting bias might be distributed similarly across the groups.

Another limitation arises from the possibility that the phenomenon of interest, participant reactivity to the study conditions (that is, their belief about whether they were in a cohort study or intervention trial, or whether they were in the control or intervention group), might be weaker in the online setting than *in vivo*. The use of the internet for experimental manipulation and data collection were necessitated by the size of the study and the need for precise control over study conditions. It may, however, be the case that research participation effects are more powerful in settings that involve direct interpersonal contact, for example, through cues transmitted in the researcher’s voice, tone, appearance or behaviour. The internet may have diminished the operation of these effects if they are driven by interpersonal processes involving performance, conformity to perceptions of researcher expectations (demand characteristics) or social desirability bias.

The near identical outcomes in reading time between the ‘cohort’ and ‘control’ groups suggest the likelihood of manipulation failure, that is, that while the ‘cohort’ group had the low level of engagement that might be expected for participants in a study merely involving measurement, the ‘control’ group, who were given to believe they were in an intervention trial, were equally uncurious about the reading materials. Reading times are probably a crude indicator of how well participants attended to web page content but measurement error is likely to be equally distributed across the randomly allocated groups.

This concern about weakness in the experimental manipulation is corroborated by the findings shown in Table 4, which indicate that the groups (B and C) who were told they were in a trial were no more likely than the ‘cohort’ group (A) at follow-up to think the study they had participated in was an intervention trial. Accordingly, hypothesis 1 might not have been adequately tested. In a subsequent experiment, we implemented a brief knowledge test in the web page following the information page to increase the likelihood that participants read and understood the text. There would be value in implementing such a procedure in a new test of this hypothesis, and also in asking participants upon completion of the follow-up some questions to investigate mechanisms, for example, ‘Did you feel that the researchers wanted you to reduce your alcohol consumption?’, ‘Did being in the study make you want to reduce your drinking?’, and ‘Did the leaflet make you want to reduce your drinking?’ In addition, after debriefing, participants could be asked what condition they thought they were in, to check whether they had read the crucial material [15].

Our experimental manipulation in relation to hypothesis 1 differs in an important way from the typical conditions in which health behaviour intervention trials are conducted. Usually participants are informed that they will be allocated to one of two or more groups and they may be blinded to the group to which they have been allocated; however, in behavioural interventions this is often not possible, for example when the effects of verbal advice are being compared with those of an information leaflet. Participants usually learn about the procedure in advance of finding out the outcome of the randomization and may experience disappointment. Exceptions to this include trials we have conducted in which participants are blind to allocation and to the fact they are in an intervention trial with a view to avoiding possible reactivity (for example, [21]). The present trial did not give participants the opportunity to feel either apprehension or disappointment about the prospect and outcome of randomization and, in retrospect, the present intervention may have been somewhat weak in activating relevant expectancies; that is, it might not have seemed sufficiently credible as either control or intervention content. Accordingly, we conclude that we have not done enough to test the possibility that the prospect of randomization might affect participant behaviour in ways that bias trial outcomes.

In relation to hypothesis 2, we have undertaken a similar experimental study by post rather than online, evaluating the possible impact of communication of randomization to either intervention or a waiting list control condition, again while delivering the same content to both groups [5]. Note that the nature of the control condition is different from that used in the present study. That study recruited participants who were already concerned about their drinking via newspaper advertisements and involved brief telephone contact with study personnel. Analysis identified some between-group differences that were consistent with the idea that participants in the waiting list control condition did what they were implicitly requested to do by researchers; namely, wait to change their behaviour. Also, unlike the present study, an active intervention understood to produce small effects was offered to both groups [5]. This comparison enhances our concern about using the internet to investigate these issues in the absence of knowledge about mechanisms, and we suggest that dedicated trials of setting effects as well as mechanistic studies are needed.

Our use of university enrolment lists as a sampling frame (rather than a simple call for volunteers) makes it possible to quantify the external validity of the trial outcomes. Previous studies of the same population show that web survey respondents have different demographic characteristics (for example, a greater proportion of women respond [22]), while late respondents tend to have poorer health behaviours, including a higher prevalence of binge drinking, than early respondents [22], suggesting that the true prevalence of such behaviours is typically underestimated. The response rate of 14% (10415/72903, Figure 1) in this study indicates that the findings might not generalize to the wider student population. However, assuming that the participants were generally more pro-social than the nonparticipants, it seems unlikely that the effects we failed to find in the study participants would be present in the nonparticipants.

So far, quantitative evidence of research participation effects is confined to the effects of assessing alcohol consumption on later self-reported drinking [23], the impacts of heterogeneous definitions of the Hawthorne effect [6], and a small and diverse group of studies examining so-called demand characteristics for a variety of behaviours with mixed results [8]. There are, however, qualitative data that suggest potentially powerful research participation effects. In interviews undertaken as part of a weight loss trial [24], participants expressed their hopes on entry to the trial, for example,

Well I was hopeful, maybe, there would be something new, if it would help me lose weight, you know, or something better than what I’ve tried before because the things I’ve done before don’t work very well.

(Participant 2 [CG], [24], p. 245)

and their disappointment upon learning that they had been allocated to a no-treatment control group, for example,

I thought I was going to get some help and nobody wants to help me. You know, and I have put on weight since the last time I came, I know I have. […] I just want someone to help me.

(Participant 6 [CG], [24], p. 245)

The challenge of investigating whether such study participant experiences affect behaviour, how pervasive are such experiences, and the extent to which intervention effect estimates are biased and in what direction, remains. The trial reported here demonstrates the feasibility of studying such phenomena on a large scale. However, key elements liable to lead to research participation effects may be missing; namely, patients with an emotional investment in what happens to them in the course of a study, and direct interpersonal contact with researchers who may unwittingly influence participant behaviour. For these reasons, we see value in mixed methods research to enhance our understanding of these issues and better identify targets for study with qualitative data. These can be combined with large-scale quantitative studies designed to measure the size of specific effects, which may be small in isolation, but large cumulatively and in synergy with each other.

## Conclusions

We found no support for either of the experimental hypotheses: (1) that knowledge of being in an intervention trial versus a cohort study would reduce subsequent alcohol consumption, and (2) that being randomly allocated to an intervention group versus a control group would reduce subsequent alcohol consumption. We are satisfied that the second of these hypotheses is safely rejected in the context of web-based behavioural intervention. In relation to the first, we doubt that our experimental manipulation allowed a robust test of the possibility that the prospect of randomization affects participant behaviour in ways that could bias trial outcomes. Personal interaction between study participants and researchers, and the kind of emotional investment in research made by patients receiving or not receiving intervention may be necessary to evoke the kinds of reactivity hypothesized by Cook and Campbell [6].

## Abbreviation

AUDIT-C: Alcohol Use Disorders Identification Test – Consumption
